# Antiviral Effect of Methylated Flavonol Isorhamnetin against Influenza

**DOI:** 10.1371/journal.pone.0121610

**Published:** 2015-03-25

**Authors:** Ahmed Abdal Dayem, Hye Yeon Choi, Young Bong Kim, Ssang-Goo Cho

**Affiliations:** 1 Department of Animal Biotechnology, Animal Resources Research Center, and Incurable Disease Animal Model and Stem Cell Institute (IDASI), Konkuk University, Gwangjin-Gu, Seoul, Republic of Korea; 2 Department of Bio-Industrial Technologies, Konkuk University, Gwangjin-Gu, Seoul, Republic of Korea; Centro de Biología Molecular Severo Ochoa (CSIC-UAM), SPAIN

## Abstract

Influenza is an infectious respiratory disease with frequent seasonal epidemics that causes a high rate of mortality and morbidity in humans, poultry, and animals. Influenza is a serious economic concern due to the costly countermeasures it necessitates. In this study, we compared the antiviral activities of several flavonols and other flavonoids with similar, but distinct, hydroxyl or methyl substitution patterns at the 3, 3′, and 4′ positions of the 15-carbon flavonoid skeleton, and found that the strongest antiviral effect was induced by isorhamnetin. Similar to quercetin and kaempferol, isorhamnetin possesses a hydroxyl group on the C ring, but it has a 3′-methyl group on the B ring that is absent in quercetin and kaempferol. Co-treatment and pre-treatment with isorhamnetin produced a strong antiviral effect against the influenza virus A/PR/08/34(H1N1). However, isorhamnetin showed the most potent antiviral potency when administered after viral exposure (post-treatment method) *in vitro*. Isorhamnetin treatment reduced virus-induced ROS generation and blocked cytoplasmic lysosome acidification and the lipidation of microtubule associated protein1 light chain 3-B (LC3B). Oral administration of isorhamnetin in mice infected with the influenza A virus significantly decreased lung virus titer by 2 folds, increased the survival rate which ranged from 70–80%, and decreased body weight loss by 25%. In addition, isorhamnetin decreased the virus titer *in ovo* using embryonated chicken eggs. The structure-activity relationship (SAR) of isorhamnetin could explain its strong anti-influenza virus potency; the methyl group located on the B ring of isorhamnetin may contribute to its strong antiviral potency against influenza virus in comparison with other flavonoids.

## Introduction

The influenza A virus, a causative agent of periodic contagious disease epidemics, is a member of the *Orthomyxoviridae* family. Influenza virus infection can lead to serious respiratory illness, respiratory complications, and high rates of mortality and morbidity, and is particularly virulent among the elderly[1].The mortality rate of the seasonal influenza virus infection in children is estimated by less than 1 per 100,000 children annually[2–4].Yearly vaccination is the primary strategy used to control influenza virus infection; however, annual vaccination has some disadvantages and limitations, including high cost, inadequate protection (especially for immunocompromised patients), and the considerable length of time required to design and produce the vaccine. Therefore, efficient control of influenza outbreaks requires the discovery and development of novel antiviral drugs [5].

There are two categories of anti-influenza drugs: the neuraminidase inhibitors such as oseltamivir (Tamiflu) and zanamivir that have been approved in many countries worldwide, while peramivir and laninamivir are approved in Japan and peramivir is approved in China and the Republic of Korea [6]. In addition, the M2 proton channel inhibitors amantadine and its derivative rimantadine that stop the infection immediately after their administration. Unfortunately, the dramatic increase in the resistance of influenza A/H1N1 against Tamiflu (influenza season of 2007 to 2008)[7], amantadine, and rimantadine [8] has incited the worldwide concern. Recently, there is an increase in the Tamiflu resistance in the clinical isolates; probably due to increase in the fitness of H275Y resistant mutant that leads to NA and HA mutations as a consequence[9,10]. Therefore, novel and safe anti-influenza drugs are a focus of drug development programs, and natural antiviral nutrients are of special interest, because they are widely available and may be used as dietary supplements to combat diseases, including influenza infection. Polyphenolic flavonoid compounds are ingested daily in the diet due to their widespread availability in fruits, vegetables, grains, tea, and wine. Dietary flavonoids have several well-established therapeutic effects and produce beneficial impacts on human health such as, immunomodulation, antibacterial, anti-fungal, anti-inflammatory, anti-oxidant, and anti-cancer activities [11,12]. Of note, there is various flavonoids showed strong anti-influenza virus property *in vitro* [13–15]. Interestingly, the flavonoids showed strong synergetic effect when with ribavirin in mice [16].The chemical structure of the flavonoids is based on the presence of a 15-carbon skeleton consisting of two benzene rings (A and B rings) connected by a heterocyclic pyran ring (ring C). Flavonoids can be classified into various classes, such as flavanones, flavonols, flavones, and others, based on the molecular substitution patterns of their carbon skeletons [17]. It is worth noting that the biological activities and health benefits of the flavonoids are attributed to their potent antioxidant effects *in vitro* and *in vivo* [18,19]. Indeed, we have reported that hydroxylation patterns play a critical role in determining the cellular functions of flavonoids [20–22]. Recently, we confirmed the anti-influenza virus potency of 3,4′-dihydroxyflavone against the influenza A virus *in vitro* and *in vivo* [23]. In addition, we recently showed that the transplantation of induced pluripotent stem cells (iPS cells) pretreated with 3,2′-dihydroxyflavone (3,2′-DHF iPS) into rats with peripheral nerve injury improved axonal regeneration and functional injury recovery in comparison with the control group [24].

Flavonols are a major flavonoid subclass that is characterized by a planar structure with a 3-hydroxyflavone backbone (IUPAC name: 3-hydroxy-2-phenylchromen-4-one). In recent years, flavonols have been a focus of research due to their important biological activities, such as antioxidant activity [25] and anti-cancer activity [26].

In this study, we compared the antiviral activities of several flavonols and other flavonoids with similar, but distinct, hydroxyl or methyl substitution patterns at the 3, 3', and 4' positions of the 15-carbon flavonoid skeleton and found that isorhamnetin, a 3′-methylated flavonol, produced a strong antiviral effect. The results of this study serve to clarify the molecular mechanism of the anti-influenza effect of isorhamnetin.

## Materials and Methods

### Ethics

All animal experiments were carried out according to the Guide for the Care and Use of Laboratory Animals and were approved by the Institutional Animal Care and Use Committee (IACUC),Konkuk University (approval no.: KU13064).

Six-week-old female C57BL/6 mice were purchased from *KOATECH* (Gyeonnggi-do, South *Korea*) and housed in filter-top cages and in specific pathogen-free animal facility at the Korea Research Institute of Bioscience and Biotechnology. The virus challenge in mice was employed using anesthesia to minimize the animal suffering. Anesthesia of mice was conducted by intramuscular injection of 40mg/kg of Zoletil50 (Virbac Laboratories, Carros, France), and 5 mg/kg of Rompun (Bayer Korea, Seoul, South Korea). The monitoring of the mice conditions was performed twice a day. We carried out the humane endpoint during the experiment of the mice survival rate. For this purpose, we euthanized using CO_2_ gas when the body weight starting to decrease to 70% of the original body weight.

### Cells, Virus, and Compounds

Madin Darby Canine Kidney (MDCK) cells were obtained from the American Type Culture Collection (ATCC CCL-3, Manassas, VA, USA). MDCK cells were routinely maintained using minimum essential media (MEM) (Gibco-BRL, Grand Island, NY, USA) supplemented with 10% fetal bovine serum (FBS, Hyclone, South Logan, UT, USA),and 100U/mL penicillin/streptomycin(Gibco-BRL)at 37°C humid incubator with a 5% CO_2_ atmosphere. The influenza A virus Puerto Rico/8/34 (H1N1) was kindly provided by the Immunogenetics Laboratory of the Department of Animal Biotechnology of Konkuk University. Before viral infection, MDCK cells were washed twice with phosphate buffered saline (PBS) and cultured in virus growth medium containing MEM without FBS, 100U/mL penicillin/streptomycin, 2 μg/mL trypsin TPCK (Sigma-Aldrich, Saint Louis, MO, USA), and 0.3% bovine serum albumin (BSA, Amresco, Solon, *OH*, USA).

Virus was serially diluted (10-fold serial dilution) and inoculated into the eggs, and then the virus titers were calculated as log10EID_50_/ml [27]. At the cellular level, virus titration was determined via the hemagglutination assay (as described in detail in the WHO surveillance guidelines [28]) and by the cytopathic effect (CPE) of MDCK cells induced by viral infection, and expressed as 50% tissue culture infectious doses (TCID_50_), which were calculated based on the method of Reed and Muench [27].In mice, 50% mouse lethal dose (MLD_50_) was defined as 50% egg infective dose (EID_50_) that led to 50% mice mortality that calculated according to Reed and Muench [27]. The flavonoids quercetin, kaempferol, isorhamnetin, diosmetin, and eriodictyol were purchased from the Indofine Chemical Company, Hillsborough, NJ, USA. The flavonoids and PD-98059 (MEK inhibitor, Calbiochem, La Jolla, CA, USA) were dissolved in dimethylsulfoxide (DMSO, Sigma-Aldrich).The final concentration of DMSO in the culture media is 0.2% (v/v). Tamiflu (Oseltamivir, 75 mg) was purchased from Roche-Korea (Seoul, South Korea) Co. Ltd.

### Cytotoxicity and antiviral assays

To determine the 50% cytotoxic concentration (CC_50_) for each of the tested flavonoid compounds, MDCK cells were seeded onto 96-well plates (NUNC, Penfield, NY, USA) at a density of 1 × 10^4^ cells per well and incubated overnight for 80% confluence. MDCK cells were washed twice with PBS and the culture medium was replaced with medium containing serially diluted flavonoids (concentrations from 10μM up to 300 μM) for 48 hr at 37°C in a 5% CO_2_atmosphere. After the incubation period, the flavonoid-containing media was replaced with new media containing 10% EZ-Cytox (Daeil Lab Service, Seoul, South Korea), and the cells were incubated in the dark for 3 hr at 37°C in a 5% CO_2_ atmosphere. The optical density was measured at 480 nm using an x-Mark spectrophotometer (Bio-Rad, Hercules, CA, USA). Cytotoxicity was estimated by comparing the cell survival rate of the flavonoids-treated cells and the DMSO-treated cells with the control (virus-untreated) cells. The control cell values were arbitrarily set as the 100% survival rate. The treated cells values were normalized to the control cell values. The calculation of CC_50_ was carried out as described in detail in S1 Text.

For determination of the antiviral activity of the flavonoids against influenza A/PR/8/34 (H1N1) infection, MDCK cells were seeded on 96-well plate and infected with 100 TCID_50_ of influenza A/PR/8/34 (H1N1) virus for 2 hr, after which the virus was removed and the cells were treated with the flavonoids at 10 μM, 50 μM, and 100 μM for 48 hr (post-treatment method). Virus-induced cell death assay were measured by calculation of the cytopathic effect (CPE) of MDCK cells induced by virus infection using MTT assay. The CPE of MDCK cells was expressed as 50% tissue culture infectious doses (TCID_50_), which were calculated based on the method of Reed and Muench [27]. The effective concentration 50 for cell death (EC_50_) was calculated (see S1 Text). Finally, the *in vitro* anti-influenza virus activity of the flavonoid was expressed as SI (selective index, CC_50_/EC_50_), which is the value of CC_50_ (50% cytotoxic concentration) in MDCK cells divided by the value of EC_50_ (50% effective concentration) against influenza A/PR/8/34 (H1N1). Additionally, we employed more anti-viral assay protocols such as, pre-treatment and co-treatment methods for determination of the mechanism of these flavonoids in the anti-viral activity against influenza virus (see S2 Text).

### Virus-induced autophagy assay

To examine the ability of isorhamnetin to block the formation of acidic vesicular organelles (AVOs) after influenza A virus infection, we used various vital staining methods for AVOs, as described previously [29]. Briefly, MDCK cells were cultured on 24-well plates until 80% confluence. Cells were infected with influenza A/PR/08/34 (H1N1) virus as described previously and treated with 50 μM of isorhamnetin using the 3 aforementioned methods of flavonoid treatment (pre-treatment, co-treatment, and post-treatment methods). Cells were stained with 5 μg/mL acridine orange (AO) (Sigma-Aldrich) and 50 μM of *monodansylcadaverine* (*MDC*) (*Sigma*-Aldrich)for 15 min at 37°C and washed 3 times with PBS, after which the cells were examined under a fluorescence microscope (Carl Zeiss, Jena, Germany) to allow detection of autophagic puncta.

Autophagy can also be measured by changes in LC3B localization, because the degree of conversion of LC3B-I to LC3B-II provides an indicator of autophagic activity. Western blotting detected LC3B as 2 bands: cytosolic LC3B-I and membrane-bound LC3B-II (which is bound to the autophagosome membrane). The molecular weight of LC3B-II is greater than that of LC3B-I. However, due to its hydrophobicity, LC3B-II migrates faster in SDS-PAGE, and therefore, has a lower apparent molecular weight.

### Viral yield reduction assay

To determine the ability of the tested flavonoid compounds to inhibit virus-induced red blood cells (RBCs) hemolysis, MDCK cells were seeded in 6-wellplates at a density of 2–3 × 10^5^ cells per well and incubated overnight until 80% confluence. Cells were washed twice with PBS and infected with the virus diluted in virus growth medium at 100TCID_50_. The virus yield reduction assay was employed after 48hr of incubation with the test flavonoids. Briefly, 50 μL of PBS was added to each well of a U-bottomed 96-well plate. The infected supernatant, with or without the test flavonoids, was serially diluted 2-fold in the previously loaded PBS. Finally, 100μL of 1% chicken RBCs was added to each well. Assays were evaluated for 45min of incubation at room temperature or until agglutination occurred. RBCs in negative wells were sedimented and formed agglutination, whereas positive wells had an opaque appearance or hemolysis with no sedimentation. Titers are presented in HA units/50μL (HAU/50μL) in comparison with the control treatment (virus without flavonoids).

### Hemagglutination inhibition (HI) assay

The hemagglutination inhibition (HI) assay was employed to test the effect of the test flavonoid compounds on virus adsorption to target cells [30]. Briefly, 50μLof the flavonoids diluted in PBS containing 0.1% BSA (at the concentrations mentioned above) was mixed with 50μL of the virus in PBS containing 0.1% BSA. After 1 hr incubation, the virus yield reduction assay was carried out to measure virus titration in a U-bottomed 96-well plate as described in the previous section. Titers are given as HA units/50μL (HAU/50μL) in comparison with the control treatment (virus without flavonoids).

### Neuraminidase inhibition (NAI) assay

The neuraminidase inhibition assay was carried out to examine the inhibitory effect of the tested flavonoids on influenza virus neuraminidase activity as described previously [31]. The flavonoid compounds were used at concentrations of 10 μM, 50 μM, and 100 μM. Tamiflu, a specific NA blocker, was used as a control with a dose of 1 μM. This assay was conducted in 96-well plates; each flavonoid concentration (50 μL) was mixed with an equal volume of the virus solution (50 μL) using reaction buffer (sodium acetate buffer 150 mM, pH 7, and 1 mM calcium chloride) and incubated for 30 minutes at 37°C. For initiation of the enzymatic reaction, we added 100 μL of the substrate solution (4-MU-NANA; (2′-(4-methylumbelliferyl)-α-d-*N*-acetylneuraminic acid, sodium salt hydrate; Sigma-Aldrich) that dissolved in the enzyme buffer [33 mM 2-(*N*-morpholino) ethanesulfonic acid (MES), pH 6.5, and 4 mM CaCl2] to the final concentration of 100 μM to the flavonoids and the virus mixture (100 μL). This reaction mixture was protected from light and incubated at 37°C for 2 hr under shaking condition. For stopping of the reaction, we added the stop solution (150 μl of 0.014 N NaOH in 83% ethanol) to each well. The fluorescence of the released 4-methylumbelliferone fluorescence was measured using a fluorescence plate reader (Molecular Devices, Sunnyvale, CA, USA) (excitation 365 nm, emission 460 nm). Based on the reported formula [31], the relative NA activities were calculated as follows:

Relative NA activity (%) = (NA_t_/ NA_v_) x100.

NA_t_: the NA activity the virus + the tested flavonoids in 0.2% DMSO.

NA_v_: the NA activity of the virus + 0.2% DMSO.

### Reverse transcription polymerase chain reaction (RT-PCR)

MDCK cells were seeded in 6-well plates and incubated overnight for 80% confluence. After the medium was removed, the cells were washed twice with PBS and infected with the virus as described in the antiviral assay section. Total RNA was isolated using Trizol (Invitrogen, Waltham, MA, USA) according to the manufacturer’s instructions. Synthesis of cDNA was performed using 5 μg of total RNA with MMLV reverse transcriptase (Promega, Madison, WI, USA). The primer sequences used in our study were as follows: HA (forward, 5′-GAAAGCTCATGGCCCAACCA-3′;reverse,5′-TCCCAGGGGTGTT-TGACACT-3′); NA (forward, 5′-TGCTTGGTCAGCAAGTGCAT-3′; reverse,5′-GGTTGTCACCGAAAACCCCA-3); ATG-5 (forward, 5′-TATCATCCCACAGCC-AACAG-3′; reverse, 5′-GACCTTCAGTGGTCCGGTAA-3′); ATG-7 (forward, 5′-ACCCAGAAGAAGCTGAACGA-3′; reverse, 5′-AGACAGAGGGCAGGATAGC-A-3′); LC3B (forward, 5′-CGGAGAAGACCTTCAAGCAG-3′; reverse, 5′-CTGG-GAGGCATAGACCATGT-3′); and GAPDH (forward, 5′-CCCATCACCATCTTCCA-GGAGC-3′; reverse, 5′-CCAGTGAGCTTCCCTTCAGC-3′).The expression of mRNA was normalized to that of the control housekeeping gene, GAPDH.

### Virus-induced ROS generation assay

For the ROS generation assay, MDCK cells were seeded onto 24-well plates and incubated overnight for 80% confluence. The MDCK cells were infected with the virus as described previously and then treated with the flavonoids (post-treatment method). Intracellular ROS levels were detected using a cell-permeable oxidant-sensitive fluorescent probe, 2′, 7′-dichiorofluorescin diacetate (H_2_DCFDA, Molecular Probes, Eugene, OR, USA). The cells were incubated with 10 μM of H_2_DCFDA for 30 min at 37°C in the dark. After incubation, the cells were washed twice and covered with PBS. The average ROS generation was determined by fluorescent images that were captured using Nikon Eclipse TE2000-U fluorescence inverted microscope (Nikon, Tokyo, Japan). In this experiment, we used N-acetyl-l-cysteine (NAC; Sigma-Aldrich), a ROS scavenger reagent [32], and at a concentration of 15 mM as a positive control treatment.

### Western blot analysis

To prepare whole-cell extracts, cells were washed 3 times using cold PBS, scraped from the dishes, and suspended in protein extraction buffer containing 1% Triton X-100 (Amresco), 100 mM Tris-HCl, 10 mM NaCl, 10% glycerol, 1 mM sodium orthovanadate (Sigma-Aldrich), 50 mM sodium fluoride (Sigma-Aldrich), and 1 mM phenylmethylsulfonyl fluoride (PMSF; Sigma-Aldrich). After incubation on ice for 30 min, lysates were centrifuged and proteins in the supernatants were quantified using the Bradford Protein Assay Reagent (Bio-Rad). Protein samples were separated via 10% SDS-PAGE and transferred to nitrocellulose membranes (0.2 mm; Protran, Newton, NJ, USA). The membranes were blocked using 5% nonfat dry milk and 0.1% Tween-20 in Tris-buffered saline and probed with the primary antibody. Western blotting was performed using antibodies against t-ERK, p-ERK, actin (Santa Cruz Biotechnology, Dallas, TX, USA), and LC3B (Novus Biologicals, San Diego, CA, USA), as well as anti-mouse, anti-rabbit, and anti-goat IgG-peroxidase-conjugated secondary antibodies (Santa Cruz Biotechnology) and an enhanced chemiluminescence (ECL) kit (Amersham Biosciences, Piscataway, NJ, USA).

### *In vivo* antiviral assay

Six-week-old female C57BL/6 mice purchased from *KOATECH* (Pyeongtaek, South *Korea*) infected with the PR8 influenza strain (A/PR/8/34, H1N1). After anesthesia, mice were inoculated with 4.25 log_10_EID_50_/mL (5 MLD_50_/mice) in 30 μL sterile PBS via the intranasal route. Two hours post-infection, each group (13 mice) received the following compounds: (1) Tamiflu capsules were dissolved in PBS at a concentration of 10mg/kg and then centrifuged at 1,000rpm/10minutes for removal of the filler. From this solution 200 μL was administered per day orally for 5 days, or (2) flavonoid (Isorhamnetin) was initially dissolved in 0.2% DMSO and then diluted in PBS to a final concentration of 1 mg/kg and less than 0.2% DMSO. This mixture mixed will by vortex and 30 μL of this mixture was administered per day via the intranasal route for 5 days.

One week after infection, the lungs of 3 mice per group were collected for the virology analysis. Lung tissues were excised, homogenized using a homogenizer (Tissue Lyser, Qiagen, Valencia, CA, USA), and centrifuged (2,000 *g* for 5 min). Ten-fold serial dilutions of the supernatant from the lung homogenate samples were subjected to the virology assays for virus titer determination. Lung homogenates were injected into the allantoic sac of 10-day-old embryonated eggs, which were incubated at 37°C for 48 hr, after which the allantoic fluid was harvested. The virus titer in the allantoic fluid was assessed using the HA assay. The virus titer (EID_50_/mL) in the fluid was calculated based on the method of Reed and Muench [33,34]. For the assessments of body weight and survival rate, mice were observed daily for 14 days and monitored for clinical signs.

### Statistical analysis

Each experiment was repeated a minimum of 3 times, and data are presented as mean ± standard deviation. For the analysis of the significance of differences, we used one-way analysis of variance (ANOVA) or the two-tailed Student's *t*-test. *P* values equal to or less than 0.05 and 0.01 were considered statistically significant.

## Results

### The antiviral potency of the methylated flavonol isorhamnetin

**Fig 1:**
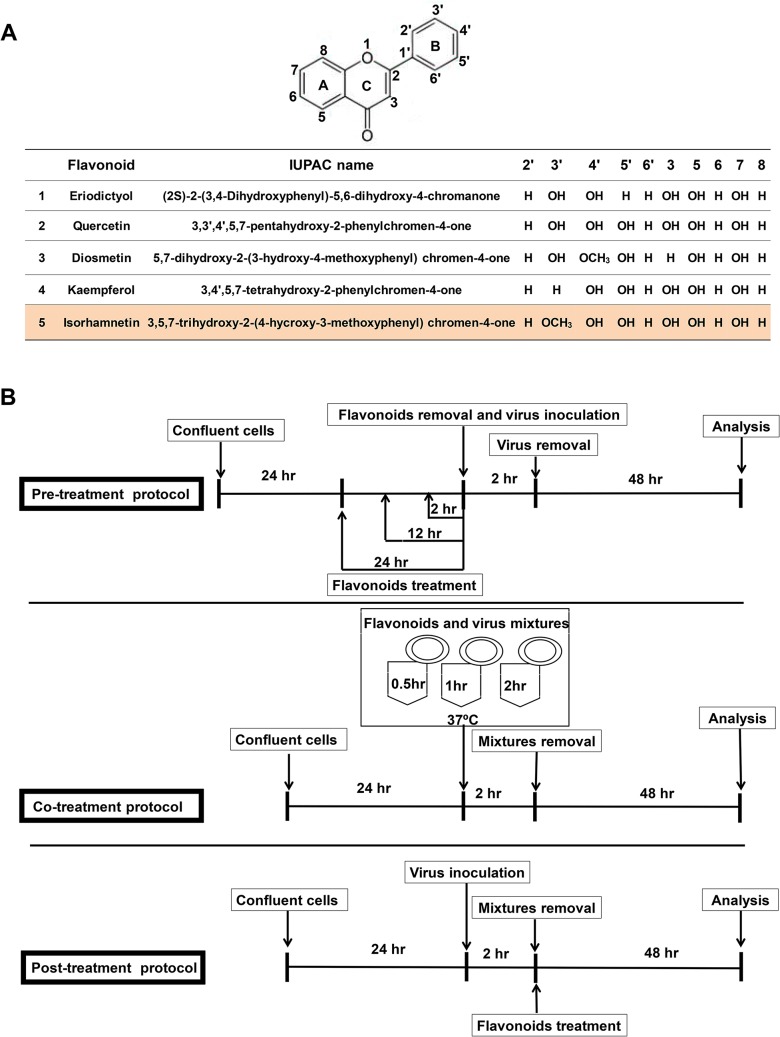
Flavonoid chemical structure, impact on cell viability, post-treatment, pre-treatment, and co-treatment effects of the flavonoids against the influenza A/PR/8/34 (H1N1) virus. (A) The chemical structures of flavonoids used in this study, showing different distributions of hydroxyl groups located on the B ring. (B) The experimental protocols for checking the antiviral activity of the tested flavonoids, which were as follows: pre-treatment, in which isorhamnetin and quercetin were administered before virus infection in a time-dependent manner (1 hr, 12 hr, and 24 hr) and a dose-dependent manner (10 μM, 50 μM, and 100 μM). Co-treatment, in which isorhamnetin or quercetin were incubated with the virus for different lengths of time during the incubation (0.5 hr, 1 hr, and 2 hr) and in a dose-dependent manner (10 μM, 50 μM, and 100 μM) before inoculation into the cells. Post-treatment, in which the flavonoids were administered after the end of the virus infection and removal of the virus.

The main goal of this study was to identify the flavonoid compound with the lowest cytotoxicity and the highest antiviral activity among the tested flavonoids, which were diosmetin, eriodictyol, kaempferol, isorhamnetin, and quercetin. These flavonoids possess 2 benzene rings (ring A and B) linked by a 3-carbon chain that forms a closed pyran ring (C ring) with variously distributed hydroxyl and methyl groups (Fig. 1A).

We investigated the influence of flavonoids treatment on the cell viability of MDCK cells. The addition of the flavonoid compounds did not produce significant cytotoxic effects in MDCK cells (Table 1).

**Table 1:** Representation of the antiviral activities of isorhamnetin and other flavonoids by SI.

**Protocol**	**Compound**	**CC**50 **(μM)**	**EC**50 **(μM)**	**SI**
**Post-treatment**	Isorhamnetin	>280	23	>12
	Quercetin	>265	30	>9
	Kaempferol	245	35	7
	Diosmetin	246	124	2
	Eriodictyol	256	115	2
**Co-treatment (0.5 hr)**	Isorhamnetin	>280	76	4
	Quercetin	>265	106	3
**Co-treatment (1 hr)**	Isorhamnetin	>280	42	7
	Quercetin	>265	62	4
**Co-treatment (2 hr)**	Isorhamnetin	>280	<30	>9
	Quercetin	>265	43	6
**Pre-treatment (1 hr)**	Isorhamnetin	>280	51	6
	Quercetin	>265	78	3
**Pre-treatment (12 hr)**	Isorhamnetin	>280	65	4
	Quercetin	>265	85	3
**Pre-treatment (24 hr)**	Isorhamnetin	>280	88	3
	Quercetin	>265	98	3

The *in vitro* anti-influenza virus activity of the flavonoid was expressed as SI (selective index, CC_50_/EC_50_), as described in Materials and Methods. Pre-treatment and co-treatment experiments were performed as described in detail in Materials and Methods.

However, diosmetin and eriodictyol treatment produced a significant decrease in the cell viability of MDCK cells, especially at concentrations of 50μM and 100μM.

For the antiviral screening, we infected the cells with the influenza virus and determined the antiviral potency of each flavonoid. To further characterize the antiviral mechanism of the flavonoids against the influenza virus, we also carried out pre-treatment and co-treatment experiments in addition to the post-treatment method, as explained in detail in materials and methods section and as illustrated in Fig. 1B.

We found that post-treatment with flavonols such as quercetin, kaempferol, and isorhamnetin led to apparent suppression of influenza virus-induced cell death, and isorhamnetin showed the strongest antiviral activity of the tested flavonoids (Table 1).

Of note, the anti-influenza virus activities of quercetin and kaempferol have been shown in previous reports [14,35]. Based on this data, we calculated the 50% cytotoxic concentration (CC_50_), the 50% effective concentration (EC_50_), and finally the selectivity index (SI) (SI = CC_50_/EC_50_). Our results revealed that isorhamnetin had the highest SI index of the tested flavonoids (Table 1).

Additionally, we evaluated the pre-treatment and co-treatment effects of isorhamnetin (50 μM) or quercetin (50 μM) on influenza virus-induced cell death (Fig. 1B).

For the pre-treatment experiment, cells were exposed to isorhamnetin (50 μM) for 1 hr, 12 hr, and 24 hr before the viral infection. Pre-treatment with isorhamnetin (50 μM) 1 hr before viral infection increased cell viability in comparison with quercetin treatment, demonstrating that isorhamnetin may have preventive effects against influenza virus-induced cell death (Table 1).

Moreover, co-treatment with isorhamnetin (50 μM) for 2 hr significantly inhibited viral infection more strongly than the other treatment durations (0.5 hr and 1 hr). Our data presented that isorhamnetin (50 μM) pre-treatment for 1 hr, co-treatment for 2 hr, and especially post-treatment produced strong and significant antiviral effects against the influenza A virus.

### Inhibition of virus-induced autophagy by isorhamnetin treatment

**Fig 2:**
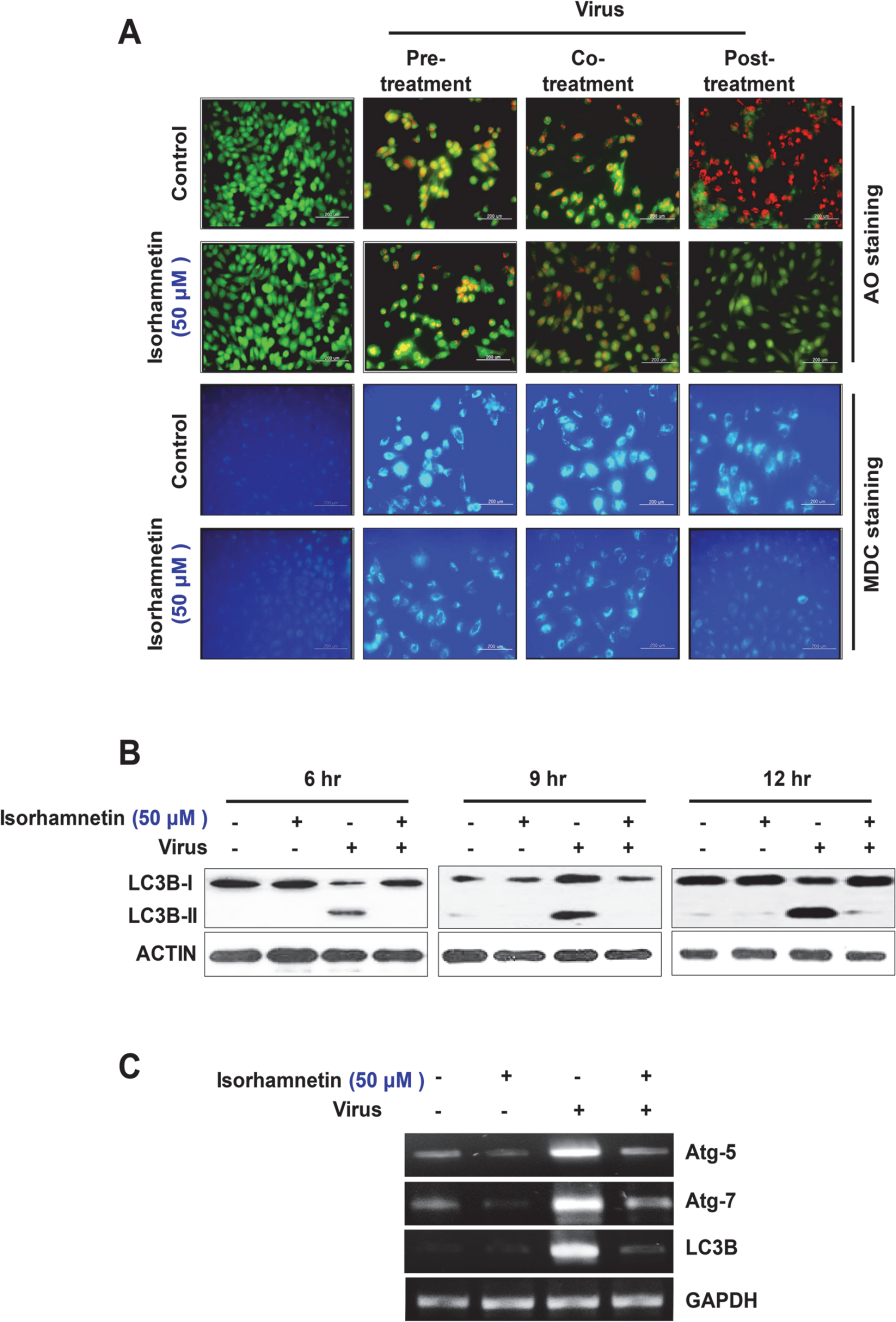
Effect of isorhamnetin treatment on the virus-induced autophagy. (A) Fluorescent microscope data for AVOs staining. MDCK cells were infected with the virus and then treated with isorhamnetin and finally stained with vital stains specific for AVOs (AO and MDC staining). (B) Western blot analysis for detection of LC3B protein lipidation. After infection with influenza A virus and treatment with isorhamnetin, we checked the lipidation of LC3B in a time-dependent manner (6 hr, 9 hr, and 12 hr). (C)RT-PCR data showing the expression level of autophagy genes. After virus infection and isorhamnetin treatment, we checked the expression level of autophagy related genes (Atg-5, Atg-7, and LC3B) using RT-PCR. Loading control = GAPDH.

Viral infection enhances autophagic flux and the lipidation of LC3B-II [36–38]. In order to examine the ability of isorhamnetin to block the formation of acidic vesicular organelles (AVOs) after influenza A virus infection, we used various vital staining methods for AVOs [29]. Our data showed that pre-, co-, and post-treatment with isorhamnetin (50 μM) significantly suppressed virus-induced autophagic puncta formation and acidification of AVOs compared with control cells (Fig. 2A).

Post-treatment with isorhamnetin (50 μM) produced a particularly strong effect, and almost completely blocked virus-induced autophagic puncta formation and AVO acidification.

Because lipidation of LC3B is considered to be a primary event that is necessary for the induction of autophagy [39] and the level of conversion of LC3B-I to LC3B-II provides an indicator of autophagic activity, we examined levels of cytosolic LC3B-Iandlipidated LC3B-II after virus infection and isorhamnetin treatment. In particular, the levels of LC3B-II correlate with autophagosome formation, due to its association with the autophagosome membrane. As previously reported [37,38] influenza virus infection significantly increased the level of lipidated LC3B-II (Fig. 2B).

Importantly, post-treatment with isorhamnetin (50 μM) significantly suppressed the lipidation of LC3B-II, confirming the strong antiviral property of isorhamnetin. Moreover, isorhamnetin (50 μM) treatment inhibited virus-induced overexpression of autophagy-related genes Atg-5, Atg-7, and LC3B (Fig. 2C).

### Inhibitory effect of isorhamnetin on virus adsorption onto RBCs

**Fig 3:**
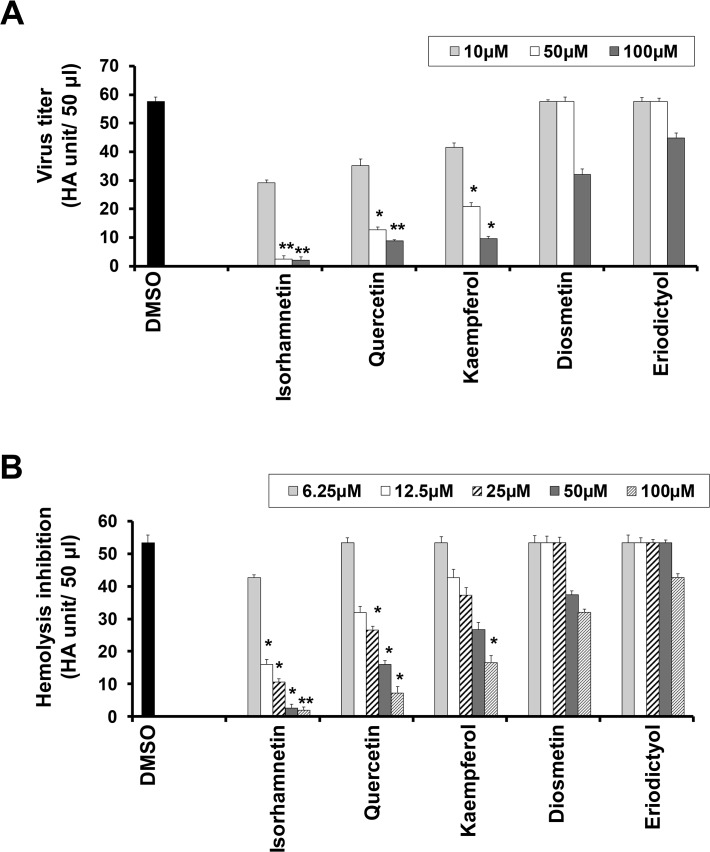
Measurement of antiviral activity of isorhamnetin by virus yield and HI assays. (A) Viral yield reduction assay was carried out by seeding MDCK cells in a 6-well plate infected with influenza A/PR/8/34 (H1N1) viruses for 2 hr, followed by virus removal, and flavonoids treatment in a dose dependent manner (10 μM, 50 μM, and 100 μM) for 48 hr. After incubation, a virus yield reduction assay was carried out using media soup. The HA titers were interpreted as HAU/50μL. *P < 0.05, **P <0.01. (B) Hemagglutination inhibition (HI) assay: The flavonoids were serially diluted using PBS and were added to an equal volume of the virus. For checking RBCs hemolysis inhibition potency, 50μL of 1% chicken RBCs were added to each well of a 96-well plate incubated for 30min at room temperature. *P < 0.05, **P <0.01.

In order to characterize the mechanism through which isorhamnetin produces anti-influenza effects, we carried out the virus yield reduction assay. Treatment with flavonols isorhamnetin, quercetin, and kaempferol significantly decreased virus titer, and isorhamnetin almost completely suppressed virus titer in HA assay (Fig. 3A).

Next, we employed the HI assay to assess the ability of the flavonoid compounds to directly interfere with virus particles. Interestingly, the lowest concentration of isorhamnetin significantly inhibited the hemolysis of chicken RBCs by the virus particle (HI assay) (Fig. 3B).

In contrast, the lowest concentration of isorhamnetin produced significant inhibition of RBCs hemolysis, but the lowest concentrations of the other tested flavonoid compounds did not have such an effect (Fig. 3B).

These results suggest that the potent HI assay activity of isorhamnetin may be attributed to its direct interaction with virus particles.

### Inhibitory effect of isorhamnetin on influenza virus NA activity and viral mRNA expression

**Fig 4:**
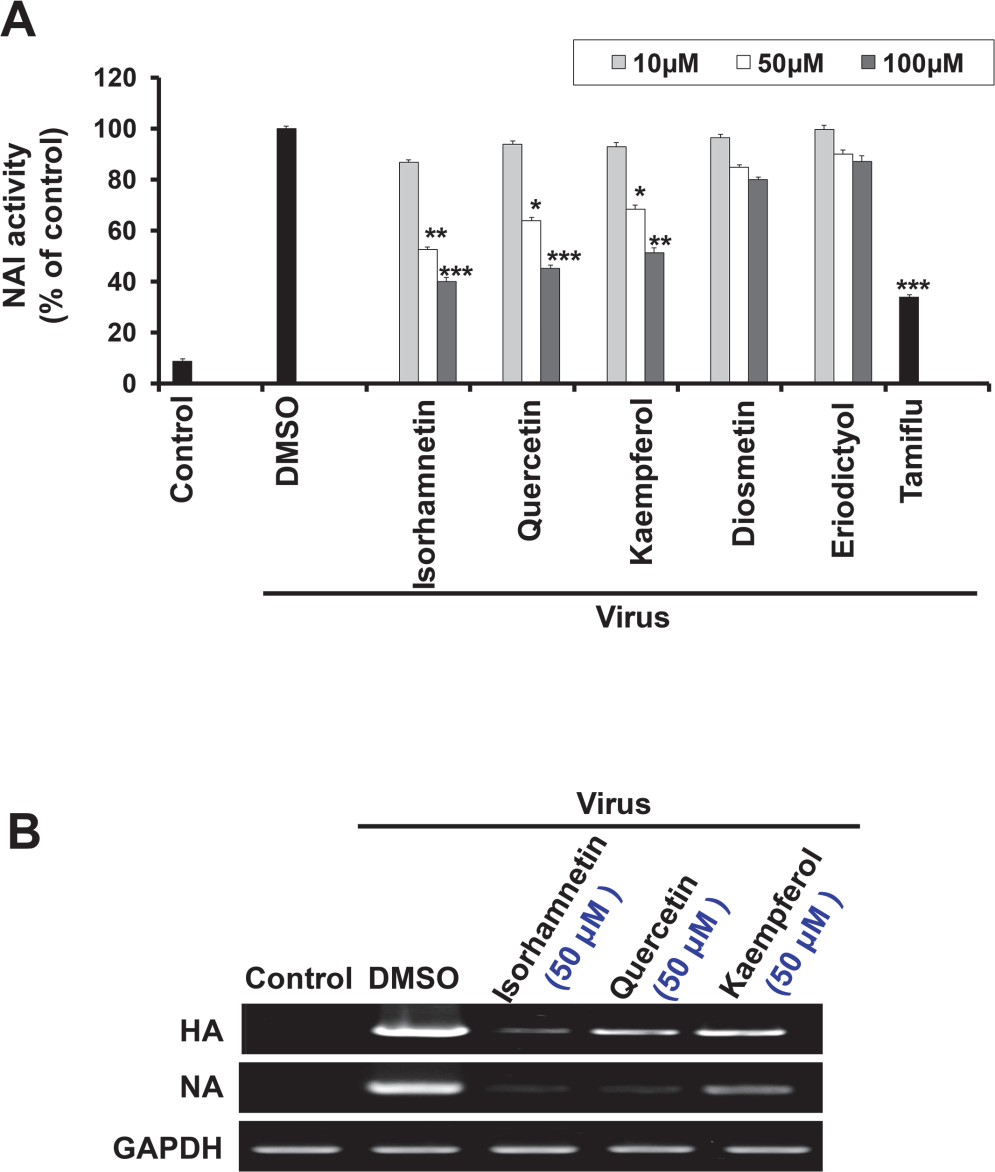
Measurement of antiviral activity of isorhamnetin by NA assay and RT-PCR. (A) NAI assay was assessed by mixing of 50 μL of the flavonoids at the indicated concentrations with 50 μL of the virus, and then adding 100μL of the substrate solution (4-MU-NANA; (2′-(4-methylumbelliferyl)-α-d-*N*-acetylneuraminicacid, sodium salt hydrate; Sigma-Aldrich) that dissolved in the enzyme buffer [33mM 2-(*N*-morpholino) ethanesulfonic acid (MES), pH 6.5, and 4 mM CaCl2].This reaction mixture was protected from light and incubated at 37°C for 2hr under shaking condition. The optical density was measured for calculating the fluorescence intensity of 4-methylumbelliferone using fluorescence spectrophotometer at excitation of 365 nm and emission at 460 nm. *P < 0.05, **P <0.01, ***P <0.001. (B) RT-PCR was carried out to detect the expression level of HA and NA genes. MDCK cells were seeded in a 6-well plate infected with influenza A/PR/8/34 (H1N1) viruses for 2 hr, followed by virus removal and flavonoids treatment in a dose dependent manner (10 μM, 50 μM, and 100 μM) for 48 hr. After incubation, RT-PCR was performed using specific primers for influenza virus HA and NA.

NA is a key viral protein that is responsible for the release of new virus particles via the recognition and cleavage of the N-acetylneuraminic acid (sialic acid moiety)receptor on the host cell membrane [40].The NAI assay revealed that neuraminidase activity was decreased after treatment with flavonols in comparison with the positive control Tamiflu(1μM). Isorhamnetin (50 μM) treatment significantly inhibited NA (Fig. 4A).

These results suggest that isorhamnetin may produce additional inhibitory effects on virus release through inhibition of NA.

Viral mRNA synthesis occurs in the middle stage of viral infection. Forty-eight hours after virus infection, we checked the expression level of influenza viral genes HA and NA. Isorhamnetin (50 μM) markedly decreased the expression of HA and NA (Fig. 4B).

### Inhibitory effect of isorhamnetin on virus-induced ROS generation

**Fig 5:**
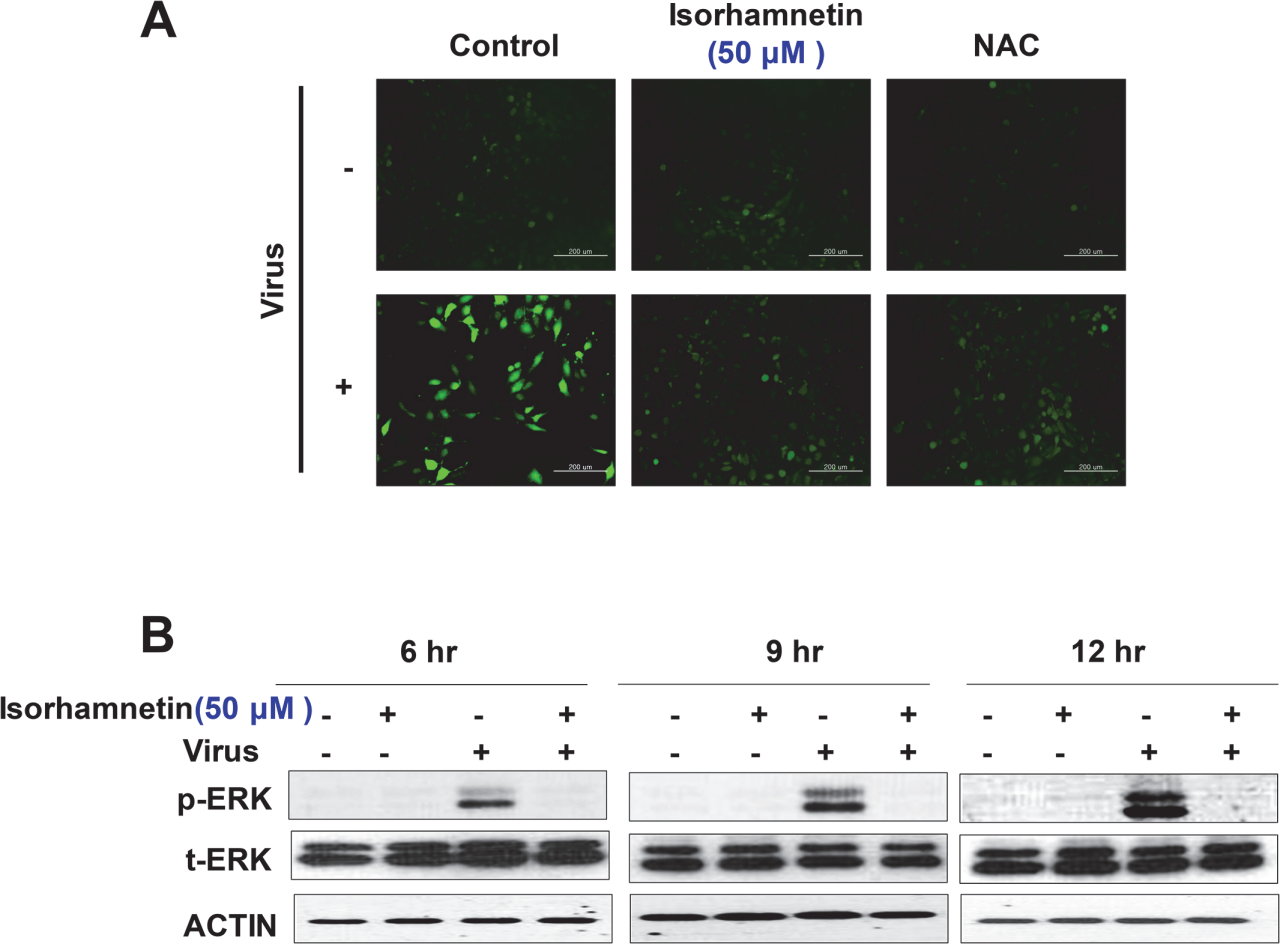
Effect of isorhamnetin on the inhibition of the influenza virus-induced ROS generation and ERK phosphorylation. (A) After influenza virus infection and isorhamnetin treatment, ROS generation was measured using ROS fluorescent probe, H_2_DCFDA. Scale bar = 200 μm. (B) Western blot analysis for phosphorylation of ERK. After influenza A virus infection and isorhamnetin treatment, we checked the phosphorylation level of ERK in a time-dependent manner (6 hr, 9 hr, and 12 hr) after virus infection (post-treatment).

We measured ROS generation in MDCK cells after virus infection and isorhamnetin (50 μM) treatment using the fluorescent ROS probe H_2_DCFDA. After influenza A virus infection, there was a significant increase in the fluorescent intensity of H_2_DCFDA. Interestingly, after isorhamnetin (50 μM) treatment there was a significant reduction in virus-induced ROS generation (Fig. 5A).

Moreover, isorhamnetin (50 μM) treatment significantly suppressed virus-induced ERK phosphorylation (Fig. 5B).

### *In vivo* antiviral activity of isorhamnetin against the influenza A virus

Mice were infected with influenza A/PR/8/34 (H1N1) and isorhamnetin was administered via the intranasal route at a dose of 1 mg/kg/day for 5 days. In addition, we used a control group of mice that were administrated Tamiflu orally at a dose of 10 mg/kg once per day for 5 days. Although previous reports showed no harmful effect of DMSO in mice at less than 1% [41–43],we checked any toxic or side effect of 0.2% DMSO in mice and confirmed no significant toxic effect form administration of isorhamnetin or 0.2% DMSO in mice (Data not shown).We compared the effect of isorhamnetin with the effect of Tamiflu against the influenza virus. The virus titers in lung tissue were measured to evaluate the inhibition of influenza virus replication. We sacrificed 3 mice per group 7 days after infection for the determination of lung viral titers, which were reported as EID_50_/mL per lung (Table 2).

**Table 2:** The *in vivo* anti-influenza virus activity of isorhamnetin.

	Virus titer EID_50_/ml (Lung tissue)	Virus titer EID_50_/ml (Embryonated chicken egg)

**PBS (W/O)**	10^5.5^	10^8.75^
**Isorhamnetin**	10^3.5^ *	10^8^ *
**Tamiflu**	10^4.5^ **	10^8.5^ *

Anti-influenza virus activity of the isorhamnetin against influenza A/PR/8/34 (H1N1)*in vivo* by determining the virus titer in embryonated chicken egg and in mice lung tissues that represented in EID^50^(50% embryo infectious dose).

*P < 0.05,

**P <0.01.

The virus titers in the lungs of the groups of mice treated with isorhamnetin and Tamiflu were 10^3.5^ and 10^4.5^ EID_50_/mL, respectively, and these values were lower than that of the control group (PBS; 10^5.5^ EID_50_/mL). Moreover, isorhamnetin treatment decreased viral titer after injection *in ovo* in the embryonated egg. The virus titers for the isorhamnetin and Tamiflu treatments were 10^8.0^ and 10^8.5^ EID_50_/mL, respectively, and these were lower than that of the control group (PBS; 10^8.75^ EID_50_/mL) (Table 2).

**Fig 6:**
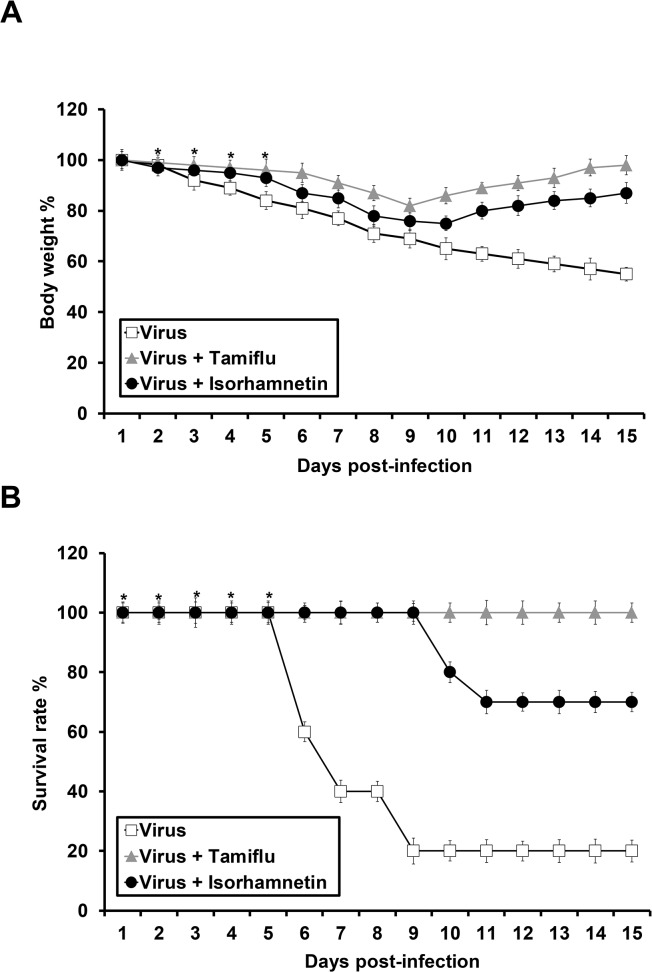
*In vivo* antiviral activity of isorhamnetin against influenza virus infection. (A)The rate of loss in body weight of six-week-old female mice(C57BL/6)after influenza A/PR/8/34 (H1N1) virus infection and isorhamnetin treatment, and Tamiflu was used as a positive anti-influenza material; *P < 0.05. (B) The survival rate in six-week-old female mice (C57BL/6) after influenza A/PR/8/34 (H1N1) virus infection and flavonoids treatment. *P < 0.05.

These data demonstrated that isorhamnetin decreased viral titer more effectively than Tamiflu. The mice were also monitored daily for 14 days to assess body weight changes and survival rates. Mice treated with isorhamnetin showed reduced body weight loss in comparison with the PBS control group (Fig. 6A).

For the survival rate, although the PBS control group of mice died by day 6after virus infection, the isorhamnetin-treated groups showed a greater survival rate, which ranged from 70–80% (Fig. 6B).

Taken together, these data confirm the *in vivo* antiviral potency of isorhamnetin against influenza A virus infection.

## Discussion

Autophagy is an essential catabolic pathway that is important in the elimination of harmful and unneeded proteins in eukaryotic cells [44]. The link between autophagy and pathogenic infections, including influenza virus infection, has been reported in previous studies [37,45]. Interestingly, autophagy is involved in the replication of the influenza virus and was associated with the lipidation of LC3B [37]. Therefore, pharmacological inhibition of influenza A virus-induced autophagy significantly reduced virus yield [37]. The exact role of isorhamnetin in the regulation of influenza A virus autophagy merits detailed further study.

Influenza virus infection resulted in significant generation of ROS and oxidative stress that was related to the release of cytokines and chemokines from the infected cells [46]. Accordingly, the use of antioxidants could be beneficial in preventing the onset or the progression of influenza. Various antioxidants, such as NAC, significantly decrease influenza virus titer via their potent antioxidant properties [47]. Therefore, pharmacological blockade of virus-mediated elevations in ROS abundance are considered to be an effective strategy with which to decrease virus titer and the inflammatory responses that are a result of influenza virus infection. Flavonoids are well-known for their antioxidant activity and excellent free radical scavenging ability, which is intimately related to their oxidation/reduction potential [48]. Our data showed that isorhamnetin treatment decreased ROS generation produced by influenza virus infection (Fig. 5A).

Recently, oligonol, a lychee fruit-derived low molecular weight polyphenol, was also reported to possess anti-influenza activity through its ability to block the attachment of the virus to MDCK cells, as well as its suppression of the nuclear export of influenza virus ribonucleoprotein (RNP) by blocking ROS-dependent induction of ERK phosphorylation[49]. The ERK signaling pathway is considered to be a main modulator of the MAPK signaling pathway, and MAPK activation during influenza virus infection has been well established [50]. Therefore, inhibition of autophagy, ROS generation, and ERK phosphorylation may be involved in the antiviral effects of isorhamnetin, a methylated flavonol.

Flavonols are an important flavonoid subclass with a planar structure that contains a 3-hydroxyflavone backbone (IUPAC name: 3-hydroxy-2-phenylchromen-4-one). During the last few decades, flavonols have been a focus of research attention due to their various and biological activities, such as antioxidant activity [25] and anti-cancer activity [26]. Quercetin is a flavonol with various biological effects related to human health, such as antioxidant activity [51], and anti-influenza virus activity [35,52]. Kaempferol is another important member of the flavonol subclass with various biological properties, such as antioxidant activity [53], and anti-influenza virus activity [14]. Isorhamnetin, a flavonol aglycone found abundantly in herbal plants such as sea buckthorn (*Hippophaerhamnoides* L.) and *Ginkgo biloba*L.has beneficial effects in the treatment of cardiovascular diseases [54], antioxidant activity [55], and protective effects against ischemic heart diseases [56]. However, there is no research report showing the antiviral activity of isorhamnetin.

To explain the biological activities of the flavonoids, structure-activity relationship (SAR) is used to assess the relationship between their chemical structures and their biological activities [57,58]. Various research groups have established the link between the chemical structures of the flavonoids and their biological activities. Previously, we revealed that eriodictyol has a protective effect against UV-induced apoptosis, which was attributed to the number and distribution of the hydroxyl groups on the carbon skeleton of the flavonoid [20]. Regarding the connection between the chemical structures of the flavonoids and their inhibitory effects on influenza virus replication, previous reports showed a correlation between the flavonoids structures and their inhibitory effects on influenza virus neuraminidases [14]. Here, our results showed that isorhamnetin has anti-influenza virus activity that is significantly stronger than that of quercetin or kaempferol. Specifically, isorhamnetin possesses a methyl group in the B ring of its carbon skeleton, emphasizing the impact of the methyl group on the biological functions of the flavonoids. Previous studies showed that methylated flavonoids are more lipophilic, and therefore these methylated flavonoids may be more readily transported through biological membranes, leading to cellular uptake better than that of the unmethylated flavonoids [59]. The flavonoids that are methylated on the B ring and A ring are reported to confer potent anti-cancer activity *in vitro* and *in vivo* [60], and another interesting report revealed that the methylated flavonoids have high hepatic metabolic stability and high intestinal absorption in comparison with the unmethylated flavonoids [61]. Methylation was reported to improve the anti-cancer potency of the flavonoids via enhancing the entry of these flavonoids into cells and inhibiting their degradation [62]. Taken together, these results suggest that the methyl group on the B ring of the carbon skeleton of isorhamnetin could be the reason that it showed the best antiviral activity among the tested flavonoids against the influenza virus.

**Fig 7:**
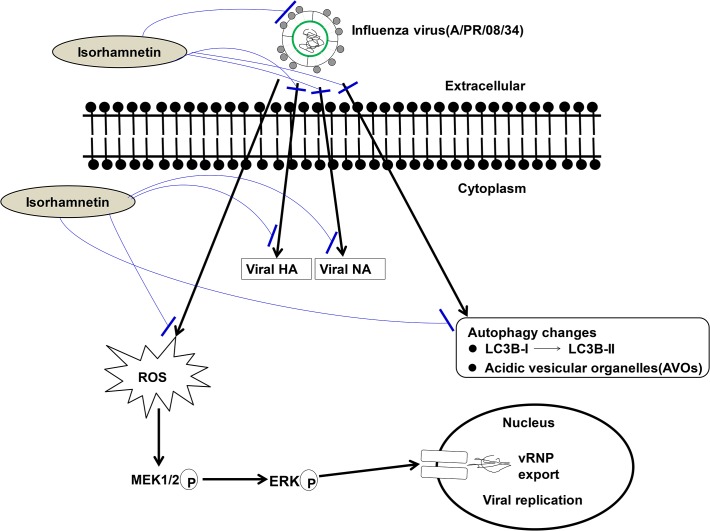
Schematic diagram represents the detailed mechanism of isorhamnetin in its antiviral activity against influenza A virus. Isorhamnetin possesses potent direct or indirect anti-influenza activity via direct suppression of virus adsorption onto host cells (HI) and NA activity (NAI) or indirect inhibition of the expression of influenza A surface proteins (HA and NA), virus-induced ROS generation and ERK phosphorylation, and the autophagic changes (AVOs formation and lipidation of LC3B) after influenza A virus infection.

Our study suggests that isorhamnetin is a natural compound with anti-influenza effects *in vitro* and *in vivo* via direct HA and NA inhibition, direct or indirect inhibition of the expression of viral HA and NA genes, and suppression of virus-induced autophagy, ROS generation, and ERK phosphorylation (Fig. 7).

## Supporting Information

S1 Text Calculation method of CC_50_ and EC_50_ for the tested flavonoids. (DOC)

S2 Text Pre-treatment and co-treatment protocols for testing the antiviral potency of the flavonoids. (DOC)
