# Supplementary material for: Antiviral Effect of Methylated Flavonol Isorhamnetin against Influenza
Source: PLoS One. 2015 Mar 25;10(3):e0121610. doi: 10.1371/journal.pone.0121610 (PMC4373826; doi:10.1371/journal.pone.0121610)
Supplement: S1 Text — (DOC) [file pone.0121610.s001.doc]

**Supporting Text Information S1**

**Calculation method of CC_50_ and EC_50_ for the tested flavonoids:**

The 50% cytotoxic concentration (CC_50_) was calculated according to the following equation:

Cytotoxicity (%) = [100-(*OD_t_*/*OD_s_*)] x100

OD_t_: The optical density (OD) of cells treated with the tested flavonoids.

OD_s_: The OD of cells treated with the control solvent (0.2 % DMSO).

After obtaining the results, we calculated the concentration that induces 50% cell death using the following equation:

(*X*2 - *X*1) X (50-*Y*1) / (*Y*2 - *Y*1) + *X*1. Where, *X*1 represents the lower concentration used in this experiment, while *X*2 represents the higher concentration. *Y*1 represents the mean of the percentage of the viable cells at *X*1 concentration. On the other hand, *Y*2 represents the mean of the percentage of the viable cells at *X*2 concentration.”

The antiviral activities of the flavonoids were calculated for obtaining the effective concentration 50 for cell death (EC_50_). EC_50_ is representing the concentration of the tested material that achieve 50% protection to the cells against virus-induced cell death . For calculation of EC_50_, we used the following equation:

The antiviral activity (%) = (*OD_t_*-*OD_v_*)/ *(OD_mock_*-*OD_v_*) X100.

*OD_t_*: The optical density (OD) of cells infected with virus and treated with the tested flavonoids.

*OD_v_*: The OD of cells that only infected with virus (virus control).

*OD_mock_*: The OD of mock-infected cells only (control cell + 0.2 % DMSO).

After obtaining the results, we calculated the concentration that induces 50% recovery of the virus-induced cell death using the following equation that described above: (*X*2 - *X*1) X (50-*Y*1) / (*Y*2 - *Y*1) + *X*1.

**References**

1. Kodama E, Shigeta S, Suzuki T, Clercq ED. Application of a gastric cancer cell line (MKN-28) for anti-adenovirus screening using the MTT method. Antiviral Res. 1996; 31: 159-164. PMID: 8811200.
