# Supplementary material for: Antiviral Effect of Methylated Flavonol Isorhamnetin against Influenza
Source: PLoS One. 2015 Mar 25;10(3):e0121610. doi: 10.1371/journal.pone.0121610 (PMC4373826; doi:10.1371/journal.pone.0121610)
Supplement: S2 Text — (DOC) [file pone.0121610.s002.doc]

**Supporting Text Information S2**

**Pre-treatment and co-treatment protocols for testing the antiviral potency of the flavonoids:**

Basically, influenza virus replication cycle can be divided into six main steps: virus adsorption, endocytosis, uncoating, packaging, budding, and finally virus release . In this context, in our study we aimed at characterization of the mechanism of the strong anti-influenza virus activity of isorhamnetin. Therefore, we carried out the pre-treatment and co-treatment methods. For the post-treatment method, this method measures the potency of the flavonoids to block the virus release via blocking of NA activity. The pre-treatment method measures the protective effect of the flavonoids to the cell receptors against the virus infection. In this method, MDCK cells were exposed to isorhamnetin or quercetin in a dose-dependent manner (10 μM, 50 μM, and 100 μM) and at different time points (1, 12, and 24 hr) before the virus inoculation. Afterwards, the media contained the flavonoids were removed, cells washed with PBS, and 100 TCID_50_ of the virus was inoculated into MDCK cells for 2 hr at 37°C. Then removing of the virus, washing with PBS, the cells were further incubated with virus growth media at 37°C for 48 hr. Co-treatment experiment measures the direct interference of the flavonoids with the virus particle binding to the cell receptors. In this experiment, which isorhamnetin or quercetin were incubated with the virus (100 TCID_50_) for different lengths of time during the incubation (0.5 hr, 1 hr, and 2 hr) and in a dose-dependent manner (10 μM, 50 μM, and 100 μM) before inoculation into the cells. After the end of each time point, we treated MDCK cells with the mixture for 2 hr. Then, after washing with PBS, we added fresh virus growth media and incubated for further 48 hr.

**References**

1. Palese P.Orthomyxoviridae: the viruses and their replication. In: Knipe DM, Howley PMeditors. Field virology. 5 ed. Philadelphia: Lippincott Williams &Wilkins; 2007.pp. 1647-1689.
